# Evaluation of Polymerization Shrinkage, Microhardness, and Depth of Cure of Different Types of Bulk‐Fill Composites

**DOI:** 10.1111/jerd.13473

**Published:** 2025-03-29

**Authors:** Sergen Özdemir, İlker Ayaz, Nurgül Çetin Tuncer, Çağatay Barutçugil, Ayşe Dündar

**Affiliations:** ^1^ Department of Restorative Dentistry Faculty of Dentistry, Akdeniz University Antalya Turkey; ^2^ Private Clinic Antalya Turkey

**Keywords:** bulk‐fill, composites, depth of cure, micro‐CT, microhardness, polymerization shrinkage

## Abstract

**Objectives:**

This study aimed to quantify polymerization shrinkage, microhardness, and depth of cure of different types of bulk‐fill composites.

**Methods:**

Six bulk‐fill composites were tested: SonicFill 2 (SCF), VisCalor (VVC), Admira Fusion x‐tra (AFX), Filtek Bulk Fill (FBF), Fill‐Up! (FUP) and GrandioSO Heavy Flow (GHF). Sixty teeth were divided into twelve groups (*n* = 5) according to the composite and the irradiation level applied (standard or high). Each tooth was subjected to three scans using a micro‐computed tomography device. The microhardness of the composite specimens was measured using a Vickers hardness tester. Depth of cure analysis was performed by measuring the microhardness of the composites at 0.5 mm intervals from the top surface to the bottom surface.

**Results:**

The highest volumetric shrinkage was found in the AFX group when polymerized at standard irradiance. After curing, the AFX group showed the highest void ratio. Increasing irradiance significantly increased volumetric shrinkage and void ratio only in the SCF group (*p* < 0.05). GHF achieved the maximum microhardness value of 80% at the lowest curing depth of 2.9 mm, while VVC, AFX, and SCF showed a curing depth of more than 5 mm (*p* < 0.05).

**Conclusions:**

The high irradiance caused the most significant volumetric shrinkage and void ratio in the SCF group. Only the FUP and GHF groups were unable to achieve the desired curing depth of 4 mm.

**Clinical Relevance:**

Irradiance, material types, and thickness are important factors in the polymerization of composites.

## 1 Introduction

The utilization of contemporary composites commenced in the early 1960s with the synthesis of bisphenol A glycidyl methacrylate (Bis‐GMA) by Dr. Rafael Bowen [1]. The numerous advantages of RBCs, including excellent aesthetics, prolonged clinical efficacy, reparability, and the safeguarding of tooth tissue during preparation due to their micromechanical bonding to tooth tissue, continue to justify their extensive usage [2]. Nevertheless, the clinical issues associated with RBCs, including marginal discoloration, cusp fractures, microleakage, secondary caries, and postoperative tenderness, warrant consideration [3, 4]. Polymerization shrinkage may be the underlying cause of these disadvantages. This shrinkage is the loss of volumetric size caused by the conversion of methacrylate monomers into polymers during the polymerization of resin‐based materials and is a structural feature of resin‐based materials. As a consequence of this volumetric dimension loss, tensile force is generated between the tooth and the restoration, which is referred to as polymerization shrinkage stress. This stress is a significant contributing factor to the failure of resin‐based restorations [5].

A variety of techniques are employed to mitigate polymerization shrinkage. These include the utilization of oblique layering during composite placement, the incorporation of stress‐absorbing intermediate layers, alterations to the light‐curing protocol, modifications to the composite formulation, and the use of bulk‐fill composites [6].

Bulk‐fill resin composites (BRBCs) have been designed to reduce the amount of polymerization shrinkage observed in conventional composites by incorporating structural modifications, namely an increase in the molecular weight of the structurally contained monomers, the addition of new stress‐relieving monomers, and methacrylate monomers with a third reactive site [7, 8]. In order to facilitate clinical use and enhance patient comfort, the BRBC can be applied to the cavity in a single layer of either 4 or 5 mm. The use of BRBCs has been demonstrated to result in a reduction in post‐gel shrinkage [9]. Furthermore, restorations utilizing bulk‐fill RBCs have been shown to exhibit a reduction in cuspal deformation, shrinkage stress, and an increase in fracture resistance when compared to conventional RBC restorations [9].

The use of composite materials, such as bulk fill, in large layers in clinical practice has prompted concerns regarding the ability to achieve an adequate depth of cure (DoC). The mechanisms employed to achieve deeper polymerization and reduce stress in bulk‐fill RBCs are distinct. Some manufacturers have endeavored to attain deeper polymerization through the utilization of supplementary or more efficacious photo initiators, such as bis‐(4‐methoxybenzoyl) diethylgermane (Ivocerin) [10]. Some manufacturers have also focused on increasing light transmission throughout the resin composite. The presence of pigments and a refractive index mismatch between the organic matrix and fillers are the primary factors responsible for the reduction in light transmission [11]. In addition to utilizing fillers and monomers with analogous refractive indices, reducing the filler content represents another strategy employed to enhance light transmission and facilitate deeper polymerization [12]. Furthermore, to address the inadequacy of adaptation to the cavity, a drawback of single‐layer application, manufacturers have introduced bulk‐fill RBCs with diverse modifications, including products applied by sonic activation or the thermo‐viscous method, or a reduced viscosity of BRBC.

A review of the literature reveals a number of studies that have examined a range of properties of bulk‐fill composites, including volumetric shrinkage, void ratios, microhardness (MH), and DoC. Nevertheless, no studies have yet evaluated bulk‐fill composites with different properties, including sonic activated, thermo‐viscous, dual‐cure, ormoser, packable, and flowable composites together. Besides, this study represents a pioneering investigation into the volumetric shrinkage evaluation with micro‐computed tomography (μ‐CT) and DoC of VisCalor (thermo‐viscous), Fill‐Up! (dual‐cure), and GrandioSo Heavy Flow (flowable bulk‐fill) composites.

A variety of experimental techniques have been developed for the measurement of polymerization shrinkage of RBCs. The majority of these methodologies calculate total shrinkage, which encompasses both pre‐gel and post‐gel shrinkage. Among the techniques employed for the assessment of volumetric polymerization shrinkage are the mercury dilatometer, water dilatometer, gas pycnometer, and buoyancy method [13].

In recent times, μ‐CT has become a popular method for measuring volumetric polymerization shrinkage. Micro‐focal point X‐ray sources and high‐resolution detectors are employed in μ‐CT systems to produce 3D reconstructed images of samples by means of projections rotated along multiple imaging directions. The non‐destructive nature of the imaging process allows for the internal properties of the same sample to be examined multiple times, retaining usability after samples have been scanned for biological and mechanical testing [14]. Samples can be examined both quantitatively and qualitatively. Volumes can be calculated using dedicated software, while specific details can be identified through visual image analysis. It has been demonstrated that this method accurately depicts the shrinkage locations of the resin composite when the adhesive is uniform and can effectively detect void formation regions and potential interface failure regions [15].

The objective of this study was to examine the volumetric polymerization shrinkage characteristics of six distinct BRBCs using μ‐CT while undergoing curing with varying light curing protocols. The null hypotheses were as follows: (1) different BRBCs exhibit similar volumetric polymerization shrinkage and void ratios, (2) the light curing protocol has no effect on volumetric polymerization shrinkage and void ratio, (3) all BRBCs have similar MH and DoC.

## 2 Materials and Methods

### 2.1 Specimen Preparations for Polymerization Shrinkage Analysis

A total of 60 sound, caries‐ and restoration‐free, permanent, freshly extracted human molars were obtained with the approval of the ethics committee numbered 2021‐ KAEK‐446, which was approved by the Akdeniz University Clinical Research Ethics Committee. The teeth were cleaned and subsequently stored in a 0.5% chloramine‐T solution at room temperature for a period of less than 3 months.

A standardized Class I preparation with a cavity design of 4.2 (± 0.5) mm diameter and 4.0 (± 0.5) mm depth was created for each tooth using a diamond disc bur and a water‐cooled high‐speed handpiece (300,000 rpm) in accordance with ISO standards. The bur was replaced after each five cavity preparations. The final cavity preparation was then checked for dimensional accuracy using a periodontal probe (Hu‐Friedy, Rotterdam, Netherlands). As a standard for restoration procedures, a universal adhesive (FuturaBond U, VOCO GmbH, Cuxhaven, Germany) was used with a selective etch technique. The enamel margins were etched using 35% phosphoric acid for 20 s, followed by a 10‐s rinse with water and subsequent drying with air. Subsequently, the adhesive system was applied to the dentin and enamel surfaces in accordance with the manufacturer's recommendations and polymerized with a light‐curing device (Valo Ultradent, USA) for 20 s in 1000 mW/cm^2^ irradiance.

In this study, six distinct bulk‐fill restorative materials were evaluated in two distinct irradiances (1000 or 1400 mW/cm^2^ irradiance). The manufacturer, composition, and lot numbers of the restorative materials utilized in the study are presented in Table 1.

**TABLE 1:** Material information of bulk‐fill resin composites used in the present study.

Material	Abbreviation	Manufacturer	Type	Content	Lot no.
Fill‐Up!	FUP	Coltene Holding AG, (Altstätten, Switzerland)	Dual‐cure bulk‐fill	Filler: (% wt.): 65, zinc oxide Matrix: TMPTMA, UDMA, Bis‐GMA, TEGDMA, benzoyl peroxide, dibenzoyl peroxide	J89970
GrandioSO Heavy Flow	GHF	Voco GmbH, (Cuxhafven, Germany)	Flowable bulk‐fill	Filler: (% wt.): 83, nanoparticles of SiO2, glass–ceramic Matrix: BisGMA, BisEMA, TEGDMA, HDDMA, CQ, amine and BHT	2006513
Admira Fusion x‐tra	AFX	Voco GmbH, (Cuxhaven, Germany)	Ormocer based bulk‐fill	Filler: (% wt.): 84.0, Ba–Al–Si‐glass/silica nanoparticles Matrix: Ormocer	2103629
Filtek Bulk Fill	FBF	3 M ESPE (St. Paul, MN, USA)	Packable bulk‐fill	Filler: (% wt.): 76.5, YbF3, zirconium, silica Matrix: Bis‐GMA, Bis‐EMA, UDMA	NA29615
SonicFill 2	SCF	Kerr (Orange, CA, USA)	Sonic‐activated bulk‐fill	Filler: (% wt.): 83.5, SiO2, glass, oxide Matrix: Bis‐GMA, TEGDMA, Bis‐EMA	8032275
VisCalor	VVC	Voco GmbH, (Cuxhaven, Germany)	Thermo‐viscous bulk‐fill	Filler: (% wt.): 83 Matrix: Bis‐GMA, aliphatic dimethacrylate	2105318

Following the bonding procedure, the teeth were randomly divided into six groups according to the materials, followed by two subgroups (*n* = 5) according to the irradiation mode. Cast filling composites were applied to the cavities in a single layer according to the manufacturer's recommendations.

### 2.2 μ‐CT Scanning

The first μ‐CT scan was performed on all teeth after cavity preparation. After the first scan, the cavities were filled with resin composite as previously described, left uncured, and immediately placed in the μ‐CT holder. To prevent unwanted curing, the μ‐CT holder was first covered with a dark plastic to avoid contact with any light source and then placed in the μ‐CT unit for the second scan and volume measurement.

Subsequently, the resin composites were cured using two different light curing protocols: standard‐1000 mW/cm^2^ irradiance (curing times of 10 s for each layer and 20 s for the final layer) or high‐1400 mW/cm^2^ irradiance (curing times of 2 × 4 s for each layer and 3 × 4 s for the final layer) with an LED curing light (VALO, Ultradent Products Inc. UT, USA) according to the manufacturer's instructions. The output of the curing unit was checked after each sample with a radiometer. Then, after all the samples were kept in distilled water for 24 h, the third and final scanning process was performed.

A high‐resolution desktop μ‐CT system (Bruker Skyscan 1172, Kontich, Belgium) was used to scan the specimens. The scanning conditions were 100 peak kilovoltage (kVp), 100 mA‐seconds (mA), 0.5 mm aluminium/copper (Al/Cu) filter, 5.2, 8.1, 11.2, and 13.74 μm pixel size, and rotation in 0.5° steps. To minimize ring artifacts, an air calibration of the detector was performed prior to each scan. Each sample was rotated 360° within an integration time of 5 min. The average scanning time was approximately 2 h. Other settings included beam hardening correction as described and the input of optimal contrast limits according to the manufacturer's instructions, based on previous scanning and reconstruction of the teeth.

The software programs NRecon (ver. 1.6.10.4, SkyScan) and CTAn (ver. 1.16.1.0, SkyScan) were used for visualization and quantitative measurements of the samples, respectively, using the modified algorithm described by Feldkamp et al. [16] to obtain axial, 2D, 1000 × 1000 pixel images. For the reconstruction parameters, ring artifact correction and smoothing were set to zero, and beam artifact correction was set to 40%. NRecon software was used to reconstruct the images obtained by the scanner to show 2D slices of the crown. In total, 512 slices were reconstructed from the entire volume. The CTAn software was also used for 3D volumetric visualization, analysis, and volume measurement of the restorations. All three scans are superimposed and perfectly aligned by the software. This procedure allowed both uncured and cured resin composite volumes to be isolated and quantified, allowing the volumetric polymerization shrinkage to be calculated as a percentage.

### 2.3 Calculation of Volumetric Change

All data obtained from the µ‐CT scans were recorded in mm³. Initially, the volume of space remaining after the adaptation of the resin composite placed in the cavity was determined. This was achieved by subtracting the volumes of the unpolymerized resin composites from the cavity volumes. Subsequently, the difference was expressed as a percentage of the cavity volume, thereby allowing the micro void ratio formed after the adaptation of the resin composite to the cavity to be recorded.

Similarly, in order to determine the volumetric shrinkage of the resin composite proportionally, the volumetric difference between the samples of polymerized and unpolymerized resin composites was proportioned to the unpolymerized volumetric values and recorded as a percentage.

Finally, the void ratio between the cavity and the polymerized resin composite was calculated as the ratio of the difference between the polymerized resin composite volume and the cavity volume to the cavity volume, expressed as a percentage.

### 2.4 Specimen Preparations for MH

To ascertain the MH of the bulk‐fill RBC material, five samples were prepared for each material using Teflon molds with a diameter of 5 mm and a thickness of either 2 or 4 mm. A Mylar strip and glass were positioned at the base of the Teflon mold, and the bulk‐fill RBCs were filled into the mold in accordance with the instructions provided by the manufacturer. Subsequently, a second Mylar strip was placed over the samples, which were then polymerized using an LED light device (Valo, Ultradent, South Jordan, UT, USA) in 1000 mW/cm^2^ irradiance for 20 s from the top surface of the samples to full contact with the strip. The samples were then stored in amber‐colored glass vials at room temperature for 24 h.

### 2.5 MH Measurement

The MH measurement was evaluated utilizing the Vickers hardness (VHN) device, which employs a square‐based MH diamond indenter. A load of 200 g was applied to the top and bottom surfaces of the samples for a period of 15 s at five different points with the indenter of the device. This resulted in five indentations being performed on each surface, with the arithmetic mean of the indentations calculated for each sample. Finally, the ratio of MH was calculated by dividing the bottom VHN value by the top VHN value for each sample, as follows: VHN ratio = (VHN bottom/VHN top) × 100, and recorded.

### 2.6 DoC Measurement

**FIGURE 1:**
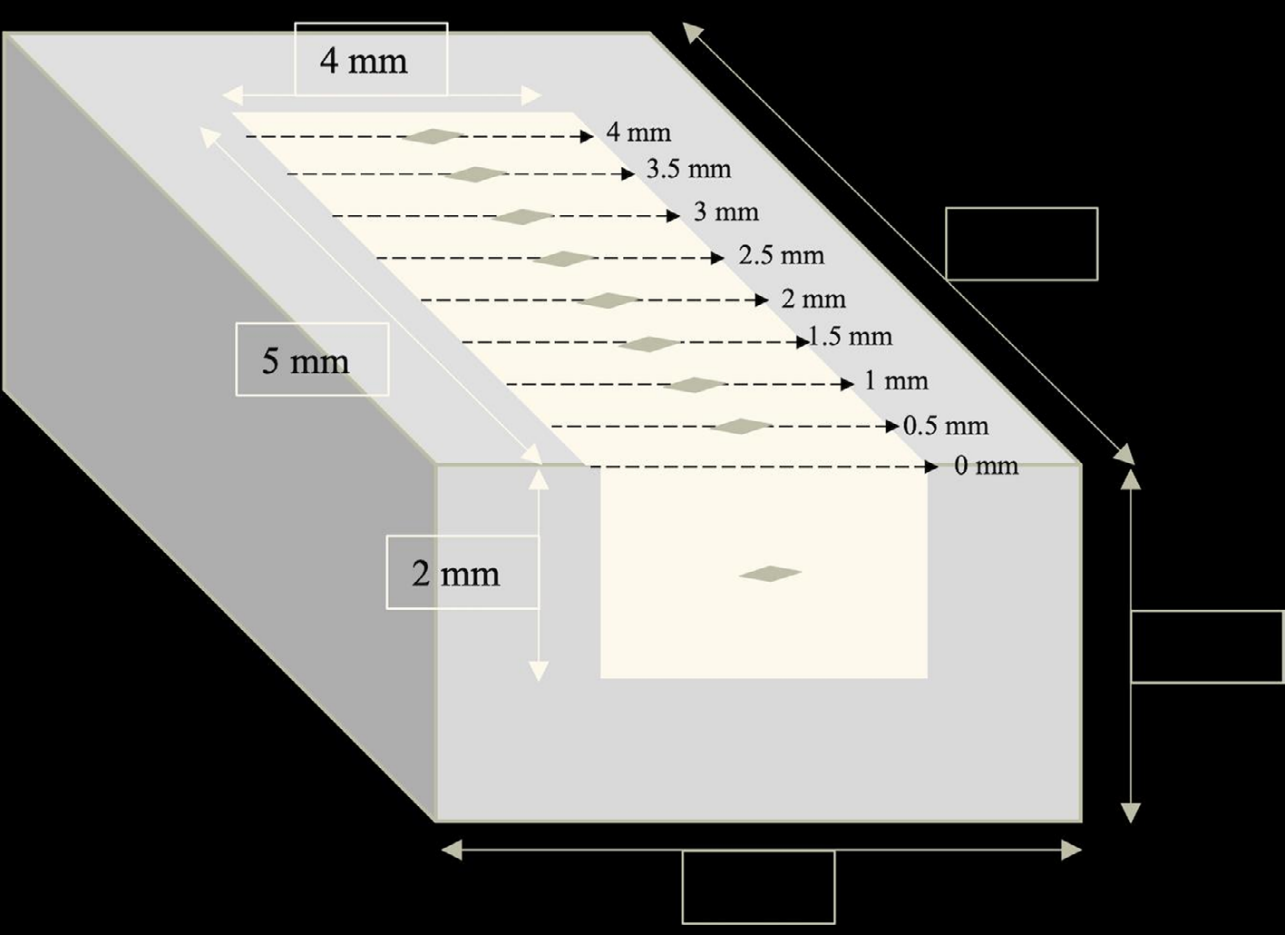
Schematic view of the Teflon mold prepared for depth of cure measurement.

The DoC evaluation was conducted in accordance with the previously reported methodology, employing the Vickers MH test. (11) Five specimens were prepared for each DoC test of the bulk‐fill RBC. The Teflon mold (dimensions of 8 mm in length, 4 mm in thickness, and 6 mm in width, with a 5 mm × 2 mm × 4 mm rectangular groove—see Figure 1) was filled with one of the RBC materials, covered with a Mylar strip after the excess material was removed, and then covered with a flat Teflon plate. A second Mylar strip was placed in the opening at the end of the mold and polymerized. Following the polymerization process, the upper Teflon layer and Mylar strips were removed, the original mold was placed within the MH apparatus, and the MH of the RBCs was measured at 0.5 mm intervals, commencing from the upper surface and progressing toward the lower layer. VHN measurements were conducted with a 50‐g load for a 10‐s period.

### 2.7 Statistical Analysis

The data obtained from the volumetric changes and MH were analyzed using the SPSS Statistics 20 for Mac program. The distributions of the values were evaluated with the Kolmogorov–Smirnov and Shapiro–Wilk tests, after which the data were analyzed with a one‐way analysis of variance (ANOVA) test and a Duncan multiple comparison test for differences between groups, as well as an independent samples *t*‐test. The significance level was determined as *p* = 0.05.

## 3 Results

The mean volumetric shrinkage values obtained with the μ‐CT are presented in Table 2. Following the polymerization process, which was conducted using standard light power, the highest volumetric shrinkage value was observed in the Admira Fusion x‐tra group, with a value of 2.1%. Conversely, the lowest value was recorded in the SonicFill 2 group, with a value of 0.6%. Furthermore, the Filtek Bulk Fill group exhibited a markedly reduced volumetric shrinkage value in comparison to the Admira Fusion x‐tra group (*p* < 0.05). No statistically significant difference was observed between the VisCalor, Fill‐Up!, and GrandioSO HeavyFlow groups and the other groups (*p* > 0.05). Upon examination of the volumetric shrinkage ratios subsequent to polymerization with high irradiance, the Filtek group exhibited the lowest ratio at 1.5%. The highest volumetric shrinkage rate was observed in the SonicFill 2 group, with a value of 2.6%. When polymerized with 1400 mW/cm^2^, the Filtek and Fill‐Up! groups exhibited significantly lower volumetric shrinkage than the SonicFill 2 and Admira Fusion x‐tra groups (*p* < 0.05).

**TABLE 2:** Volumetric shrinkages of bulk‐fill resin composites.

	FUP	GHF	AFX	FBF	SCF	VVC
Standard irradiance	1.2 ± 1.0 (ab)	1.2 ± 0.8 (ab)	2.1 ± 0.7 (b)	1.1 ± 0.4 (a)	0.6 ± 0.6 (a,A)	1.8 ± 0.7 (ab)
High irradiance	1.5 ± 1.1 (a)	1.9 ± 0.9 (ab)	2.6 ± 1.3 (b)	1.5 ± 0.8 (a)	2.6 ± 1.4 (b,B)	2.2 ± 0.7 (ab)

*Note*: The lowercase letters in the rows indicate statistically different groups according to the one‐way analysis of variance and Duncan Multiple Comparison test results (*p* < 0.05). Uppercase letters in the columns indicate statistically different groups according to the Independent *t*‐Test results (*p* < 0.05).

The mean void values following the adaptation of the composites to the cavity, as determined by μ‐CT measurements, are presented in Table 3. Among the standard light power groups, the Fill‐Up! group exhibited the highest void ratio, at 1.3%, following adaptation to the cavity. The VisCalor group exhibited the lowest void values, at 0.6%, which were statistically lower than those observed in the Filtek and Fill‐Up! groups (*p* < 0.05). No statistically significant difference was identified between the SonicFill 2, Admira Fusion x‐tra, and GrandioSO groups and the other groups (*p* > 0.05). Upon examination of the void ratios subsequent to polymerization with 1400 mW/cm^2^ irradiance, it was observed that the Fill‐Up! group exhibited the highest void ratio at 1.3%. Conversely, the VisCalor group demonstrated the lowest void ratio at 0.6%. According to the statistical analysis, the only significant difference was observed between the VisCalor and Fill‐Up! groups (*p* < 0.05).

**TABLE 3:** Void ratio after adaptation of bulk‐fill resin composites to cavity.

	FUP	GHF	AFX	FBF	SCF	VVC
Standard irradiance	1.3 ± 0.5 (b)	1,0 ± 0.8 (ab)	0.9 ± 0.4 (ab)	1.3 ± 0.8 (b)	0.8 ± 0.5 (ab)	0.6 ± 0.4 (a)
High irradiance	1.3 ± 0.9 (b)	1.1 ± 0.9 (ab)	0.9 ± 0.4 (ab)	1.2 ± 0.9 (ab)	0.8 ± 0.4 (ab)	0.6 ± 0.6 (a)

*Note*: The lowercase letters in the rows indicate statistically different groups according to the One Way Analysis of Variance and Duncan Multiple Comparison test results (*p* < 0.05). Uppercase letters in the columns indicate statistically different groups according to the Independent *t*‐Test results (*p* < 0.05).

**FIGURE 2:**
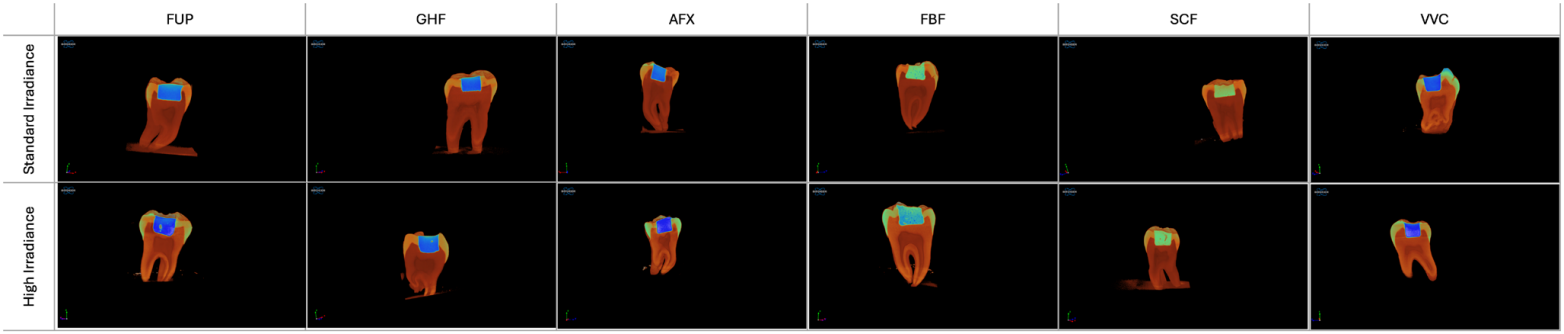
Representative μ‐CT images of teeth after light‐curing bulk‐fill composites.

The mean void values obtained with the μ‐CT after light curing are presented in Figure 2 and Table 4. Upon analysis of the samples that polymerized with 1000 mW/cm^2^ irradiance, it was observed that the Admira Fusion x‐tra group exhibited the highest void ratio, at 3.1%. The lowest void ratio was observed in the SonicFill 2 group, with a value of 1.5%. The statistical analysis revealed that SonicFill 2 exhibited a significantly lower void ratio in comparison to the VisCalor, Admira Fusion x‐tra, and Fill‐Up! groups (*p* < 0.05). No statistically significant difference was observed between the Filtek Bulk Fill and GrandioSO groups and the other groups (*p* > 0.05). With regard to the void ratios observed following polymerization with 1400 mW/cm^2^ irradiance, the highest value was recorded for the Admira Fusion x‐tra group (3.6%), while the lowest was noted for the Filtek Bulk Fill group (2.7%). Upon examination of the void ratios following polymerization with 1400 mW/cm^2^ irradiance, no significant difference was evident between the groups (*p* > 0.05).

**TABLE 4:** Void ratio between cavity and polymerized bulk‐fill resin composites.

	FUP	GHF	AFX	FBF	SCF	VVC
Standard irradiance	2.6 ± 1.0 (b)	2.3 ± 1.4 (ab)	3.1 ± 0.8 (b)	2.4 ± 0.8 (ab)	1.5 ± 0.9 (a, A)	2.4 ± 1.1 (b)
High irradiance	2.9 ± 0.8	3.1 ± 1.7	3.6 ± 1.2	2.7 ± 0.9	3.5 ± 1.7 (B)	2.8 ± 0.8

*Note*: The lowercase letters in the rows indicate statistically different groups according to the One Way Analysis of Variance and Duncan Multiple Comparison test results (*p* < 0.05). Uppercase letters in the columns indicate statistically different groups according to the Independent *t*‐Test results (*p* < 0.05).

The statistical analysis revealed that, in the SonicFill 2 group, polymerization with high light irradiance resulted in significantly greater volumetric shrinkage than polymerization with standard light output (*p* < 0.05). Conversely, in other resin composites, the polymerization mode did not exert a significant influence on volumetric shrinkage (*p* > 0.05). (Table 2) In the comparison of void ratios after adaptation to the cavity, no significant difference was found between the groups that polymerized with standard light and high light output (*p* > 0.05) (Table 3).

The data revealed that, in the SonicFill 2 group, the polymerization with high light output resulted in a significantly higher incidence of void formation in comparison to the polymerization with 1000 mW/cm^2^ irradiance (*p* < 0.05) (Figure 1). However, no significant effect of polymerization with different light outputs on void formation was observed in any of the other resin composite groups (*p* < 0.05) (Table 4).

### 3.1 MH

The lowest VHN_max_ values were observed on the top and bottom surfaces of the 2 mm thick samples in the FUP group, while the highest VHN_max_ values were observed in the GHF group (*p* < 0.05). Similarly, in the 4 mm thick samples, the lowest VHN_max_ values were observed in the FUP group. The highest values were observed in the GHF group on the top surface and in the VVC group on the bottom surface (*p* < 0.05).

In terms of curing depth, the VHN ratio values for the FUP group (80.9 ± 4.5) exhibited the lowest ratio in the 2 mm samples, which was statistically significant (*p* < 0.05). In the 4 mm samples, the lowest values were observed in GHF (54.6 ± 7.4), while the highest values were observed in VVC (96.3 ± 2.0) and AFX (94.9 ± 4.1), respectively. These findings were statistically significant (*p* < 0.05) (Table 5).

**TABLE 5:** Mean and standard deviation of microhardness results (VHN) of the groups.

	VHN_Max_ 2 mm		VHN_Max_ 4 mm		VHN_Bottom/Top_ (%)	
	Top	Bottom	Top	Bottom	2 mm	4 mm
FUP	46.2 ± 2.1 (a)	37.2 ± 2.6 (a)	43.1 ± 0.7 (a)	31.5 ± 2.2 (a)	80.9 ± 4.5 (a)	73.3 ± 5.9 (b)
GHF	72.3 ± 4.1 (e)	68.8 ± 4.9 (e)	73.2 ± 3.7 (e)	39.6 ± 3.7 (b)	95.2 ± 2.4 (b)	54.6 ± 7.4 (a)
AFX	52.9 ± 0.8 (b)	52.1 ± 1.0 (b)	53.0 ± 3.5 (b)	50.1 ± 1.9 (c)	99.2 ± 3.3 (b)	94.9 ± 4.1 (c)
FBF	53.4 ± 3.1 (bc)	52.1 ± 1.7 (b)	56.2 ± 1.1 (bc)	49.4 ± 1.2 (c)	97.9 ± 3.2 (b)	88.1 ± 1.5 (c)
SCF	58.1 ± 2.2 (c)	57.6 ± 1.7 (c)	58.8 ± 3.3 (c)	51.7 ± 3.2 (c)	99.4 ± 2.0 (b)	88.4 ± 5.5 (c)
VVC	65.9 ± 1.4 (d)	63.7 ± 1.5 (d)	65.9 ± 1.2 (d)	63.4 ± 2.3 (d)	96.7 ± 1.4 (b)	96.3 ± 2.0 (c)

*Note*: According to the results of one‐way analysis of variance and Tukey HSD multiple comparison test. Lowercase letters in the columns indicate statistical differences between the bulk‐fill RBC materials used (*p* < 0.05).

### 3.2 DoC

**FIGURE 3:**
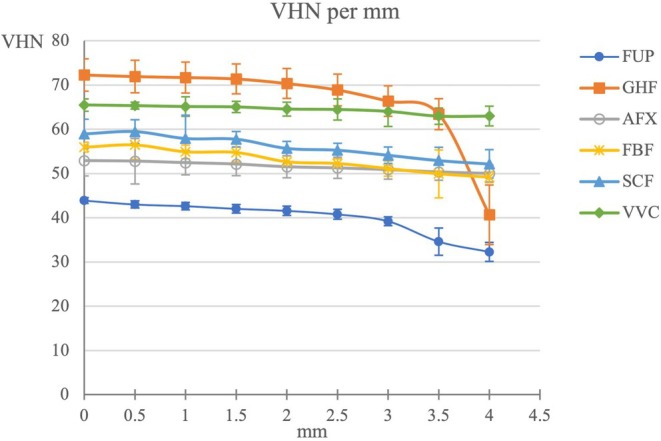
Non‐linear plots of microhardness values at measured depths for each material.

In terms of the depth at which the maximum 80% MH value was determined, GHF showed the lowest value of 2.9 (0.2) mm, and VVC (5.7 ± 0.6 mm), AFX (5.1 ± 1.2 mm), and SCF (5.0 ± 0.6 mm) showed the highest value (*p* < 0.05) (Table 6). In addition, non‐linear plots of the MH values determined at the measured depths for each material are shown in Figure 3.

**TABLE 6:** Mean and standard deviation of depth of cure results.

	VHN_Max_	Depth at 80% of VHN (mm)
FUP	43.9 (0.7) (a)	3.4 (0.3) (ab)
GHF	72.3 (3.7) (e)	2.9 (0.2) (a)
AFX	52.9 (3.5) (b)	5.1 (1.2) (c)
FBF	55.9 (1.1) (bc)	4.8 (0.9) (bc)
SCF	58.9 (3.3) (c)	5.0 (0.6) (c)
VVC	65.5 (1.4) (d)	5.7 (0.6) (c)

*Note*: According to the results of one‐way analysis of variance and Tukey HSD multiple comparison test. Lowercase letters in the columns indicate statistical differences between the bulk‐fill RBC materials used (*p* < 0.05).

## 4 Discussion

In the present study, six distinct BRBCs were polymerized with varying light power levels, and the resulting volumetric polymerization shrinkage and microvoid values were evaluated. Furthermore, MH and DoC were also investigated with a standard irradiance. Upon examination of the volumetric polymerization shrinkage and void values of the tested bulk‐fill RBCs, statistically significant differences were identified among the bulk‐fill RBCs (*p* < 0.05). Consequently, the first null hypothesis, which proposed that different BRBCs would exhibit similar volumetric polymerization shrinkage and void ratios, was rejected.

In a study conducted by Sampaio et al. [17] the volumetric polymerization shrinkage of four distinct flowable bulk‐fill RBCs was evaluated through the use of μ‐CT. The volume of each sample was measured on three occasions: initially following the preparation of the cavity, then following the filling of the cavity with composite prior to curing, and finally after the completion of the curing process. The notable discrepancies (*p* < 0.05) observed between the bulk‐fill RBCs were consistent with the findings presented in this study. In a separate investigation, Sampaio et al. [18] filled the class 1 cavities with five distinct bulk‐fill RBCs and observed the lowest volumetric shrinkage in Tetric Evoceram Bulk Fill and Filtek Bulk Fill. The finding that the lowest volumetric shrinkage rate was noted in the Filtek Bulk Fill group during polymerization with high light power in our study corroborates the aforementioned study in this regard. A high molecular weight modified urethane dimethacrylate (AUDMA, aromatic urethane dimethacrylate) has been incorporated into the composition of Filtek Bulk Fill. The manufacturer asserts that by modifying the proportions of high molecular weight monomers, Filtek Bulk Fill will exhibit reduced polymerization shrinkage, leading to diminished stress [19]. The observed low volumetric shrinkage may be attributed to the presence of high molecular weight monomers.

In a separate investigation, the volumetric shrinkage of one hybrid, three flowable, and two condensable bulk‐fill RBCs was assessed via μ‐CT. The lowest volumetric shrinkage was observed in the SonicFill 2 group, with a value of 1.2% [20]. This can be attributed to the fact that the SonicFill 2 group has the highest filler content. The extent of polymerization shrinkage is contingent upon the mobility, molecular weight, and functionality of the monomers [21]. The percentage of inorganic filler influences fluidity and viscosity; thus, a higher filler content is anticipated to result in diminished volumetric shrinkage. Moreover, Junior et al. [22] conducted a comparative analysis of the volumetric shrinkage of bulk‐fill RBCs, with the lowest shrinkage observed in the SonicFill 2 group. Similarly, in the present study, the lowest volumetric shrinkage rate among the groups was observed for SonicFill 2 when resin composites were cured with standard light power.

There are very few studies in the literature on the volumetric change after polymerization about Fill‐Up! and Admira Fusion x‐tra bulk‐fill RBCs. In a study conducted by Grenier et al. [23], the post‐polymerization volumetric change of bulk‐fill RBCs was measured, and it was reported that the shrinkage rate was higher in Fill‐Up! than in SonicFill 2 and Filtek One Bulk Fill Restorative RBCs. This may be attributed to the incorporation of the novel crosslinking agent, trimethylolpropane trimethacrylate (TMPTMA), in the Fill‐Up! formulation [23]. TMPTMA is a low‐viscosity, trifunctional monomer that exhibits the capacity to function as a crosslinking agent in polymeric matrices. Rizzante et al. [24] compared the volumetric polymerization shrinkage values of bulk‐fill RBCs and found the lowest value in the Admira Fusion x‐tra group after x‐tra fill. On the contrary, in the present study, the highest shrinkage was observed in the Admira Fusion x‐tra group after polymerization with standard light power. The reason for this difference may be related to the fact that the post‐polymerization volume measurement of bulk‐fill RBCs in their study was performed immediately after curing, rather than 24 h. Because it is known that the polymerization and transformation of resin composites continues up to 24 h after light‐curing [25].

Undesirable voids within resin‐based composites can negatively impact their mechanical properties [26]. Internal voids in the RBC mass may reduce its durability, leading to fractures and failures in dental restorations [27]. Demirel et al. [28] utilized a μ‐CT device to evaluate the void ratios of condensable bulk‐fill RBCs (SonicFill 2, VisCalor Bulk, Filtek One Bulk Fill Restorative, and Tetric EvoCeram Bulk Fill) and a conventional posterior RBC (Clearfil Majesty Posterior). Their findings indicated that, except for SonicFill 2, all resin composites exhibited a higher void formation when the sonic activation method was applied compared to other methods (*p* < 0.05). Moreover, in all resin composites except SonicFill 2, lower void ratios were observed when the pre‐heating method was employed. In the present study, although placement methods were not directly compared, the VisCalor group, which was applied using the pre‐heating method, exhibited the lowest void formation after adaptation to the cavity.

In a previous study, conventional resin‐based composite, glass ionomer, and four bulk‐fill RBCs were evaluated to investigate polymerization shrinkage stress. Among these materials, Fill‐Up! exhibited the highest internal void formation ratio [29]. This may be attributed to its rheological properties, weak interaction between the adhesive and dentin, and high polymerization stress. Since the self‐cure mode initiates polymerization immediately after mixing the base and catalyst pastes, the premature formation of isolated polymerization cores may hinder proper contact between the material and the cavity walls [30]. Similarly, in the present study, the highest void formation ratio after cavity adaptation was observed in the Fill‐Up! group.

Our results indicated that higher volumetric shrinkage and void ratios were observed in high light‐power polymerization compared to standard light‐power polymerization. Accordingly, the second null hypothesis, which stated that the light‐curing protocol had no effect on volumetric polymerization shrinkage and void ratio, was rejected. Among the tested materials, the Filtek Bulk Fill group exhibited the lowest void ratio following polymerization with high light power.

In a previous study, Demirel et al. [31] investigated void formation using μ‐CT in one conventional condensable, one conventional flowable, two flowable bulk‐fill, and one condensable BRBC. Their findings demonstrated that Filtek One Bulk Fill had the lowest microvoid ratio. This may be attributed to its slight condensation upon placement in the cavity. It is well established that applying pressure to a polymer material in its plastic state can significantly reduce void formation [32]. Furthermore, using this material in a single‐layer application can help prevent the formation of gaps between layers.

The light power density applied is also one of the factors influencing the mechanical properties of the RBC. In their study, Besegato et al. [33] cured the three different BRBCs with VALO Cordless (Ultradent Products, South Jordan, UT, USA) in a standard power mode protocol (Sp) (1000 mW/cm^2^) and an Xtra mode protocol (Xp) (3200 mW/cm^2^). They investigated the effect of light power density on polymerization shrinkage using μ‐CT. For all resin composites analyzed, the volumetric polymerization shrinkage observed in the Xp mode was higher than in the Sp mode. Irradiation exposure of 3200 mW/cm^2^ may have accelerated the polymerization reaction, reduced the viscoelastic flow of the bulk‐fill RBC, and consequently caused more shrinkage during curing [34, 35]. Over a longer period of time, low power irradiation results in slower polymerization compared to high power irradiation, which improves the mechanical properties of resin composites by forming longer chains with higher molecular weights [36]. Again, in the present study, all bulk‐fill RBCs showed greater volumetric polymerization shrinkage when polymerized at higher light power. In this respect, the results of both studies are similar.

In another study, the effect of light power density on volumetric polymerization shrinkage was evaluated by polymerizing four different RBCs at high (1200 mW/cm^2^) and low (650 mW/cm^2^) light powers [37]. There was no significant effect of applying different light powers on volumetric polymerization shrinkage (*p* > 0.05). In this study, significantly more volumetric polymerization shrinkage was observed when high light intensity was applied only in the SonicFill 2 group (*p* < 0.05). Except for SonicFill 2, no significant effect of polymerization at different light levels on volumetric polymerization shrinkage was found in any of the bulk‐fill RBCs (*p* > 0.05).

An interesting study was also carried out by Almeida et al. [38] They investigated whether there is a correlation between volumetric polymerization shrinkage and void formation phenomena in bulk‐fill RBCs applied to Class 1 cavities and evaluated by μ‐CT. They found moderate and weak positive correlations between polymerization shrinkage and void formation, respectively. Regression analysis showed that 94.5% of the void volume increased, indicating that the final void volume was due to polymerization shrinkage. This can be explained by the idea that molecular rearrangement of monomers in a smaller area and polymerization shrinkage forces can increase the void around this area [39]. In our study, when the light power level was increased, both the void ratio and the volumetric polymerization shrinkage ratio increased for all bulk‐fill RBCs. However, there is a multifactorial relationship between these two parameters. The relationship between polymerization shrinkage and void formation needs to be supported by further studies.

The MH findings of this study showed that material type and sample thickness have a significant effect on these parameters. In 2 mm thick specimens, the lowest VHN_max_ values were observed on the top and bottom surfaces of the FUP group, which is a dual‐cure composite, while the highest VHN_max_ values were found in the GHF group, which is a flowable bulk‐fill composite. In 4 mm thick specimens, the lowest VHN_max_ values were again found in the FUP group, while the highest values were recorded in the GHF group on the top surface and in the VVC group with pre‐heating treatment on the bottom surface. The preheating process is claimed to increase the mobility of the growing chains of the VBF, gradually increasing the crosslink density until it reaches the gel point, which improves the mechanical properties of the material [40].

In terms of polymerization depth, the lowest VHN ratio (80.9% ± 4.5%) was found in the FUP group in 2 mm samples, while the lowest value was found in the GHF (54.6% ± 7.4%) group in 4 mm samples, and the highest values were found in the VVC (96.3% ± 2.0%) and AFX (94.9% ± 4.1%) groups, respectively.

The most important factor in the VHN_max_ value of FUP being considerably lower than the other composites may be the lower filler content (65%). The low hardness of the 4 mm thick substrate can be attributed to the dual cure polymerization mechanism of the composite and the presence of photoinitiator and TMPTMA monomer. Both the monomer composition and the type and amount of the photoinitiator significantly influence the cure depth of RBCs, as the efficiency of each photoinitiator in generating free radicals and initiating the polymerization reaction varies [41, 42, 43].

GHF bulk‐fill material was found to demonstrate the highest hardness in the top layer and the lowest hardness in the bottom layer at a thickness of 4 mm. Furthermore, the depth at which the material reaches 80% of its maximum hardness was determined to be 2.9 mm, which is the lowest value observed among the materials. This is far below the promised depth of 4 mm for bulk‐fill composites. It is noteworthy that the MH value starts to decrease after a depth of about 2 mm, and there is a sudden decrease in MH especially after 3.5 mm (Figure 3). The polymerization depth of bulk‐fill composites has been shown to be directly related to light transmittance, with the lower MH value of the GHF group on the bottom surface attributable to the inverse proportionality of light transmittance to material thickness. Rizzante et al. [24] have reported that the filler content and monomer structure of bulk‐fill composites are determinants of polymerization depth. The type and distribution of filler particles utilized in the GHF group may have restricted light penetration, consequently yielding a reduced MH value on the bottom surface.

A previous study indicated that bulk‐fill composites can often be applied at a depth of 4–6 mm [44]. However, the present study shows that there are distinct differences between different bulk‐fill materials, with some materials offering lower MH in deeper layers. In particular, further investigation should be carried out for the factors limiting the polymerization depth of the GHF group. In this study, it was observed that all bulk‐fill composites except FUP and GHF exceeded the application thickness of 4 mm.

The third null hypothesis, which states that all BRBCs possess analogous MH and DoC, is refuted upon thorough evaluation of the available data on MH and DoC. Further research is required to investigate the light transmission of the materials in different thicknesses, the polymerization mechanism by various quantitative methods (FTIR, Raman spectroscopy), and in vivo studies. Such research may support the results of the present study.

## 5 Conclusion

In the context of the study's limitations, which pertained to the investigation of volumetric polymerization shrinkage, void ratio, MH, and DoC of BRBCs, the ensuing results have been determined.

1. High light polymerization resulted in greater volumetric polymerization shrinkage compared to standard light polymerization in all BRBCs. However, this increase was significant only in the SCF group.
2. The highest volumetric shrinkage rate after polymerization with standard curing light was found with AFX, and the lowest rate was found with SCF. After curing at high light intensity, the highest volumetric shrinkage rate was found in the SCF group, and the lowest rate in the FBF group.
3. After curing, the AFX group showed the highest void ratio. Increasing irradiance significantly increased volumetric shrinkage and void ratio only in the SCF group.
4. A significant variation in MH values was observed among the different groups, with the lowest values recorded in the FUP group and the highest in the GHF, VVC, and AFX groups, depending on thickness and surface.
5. The DoC analysis revealed that GHF exhibited the lowest curing depth, while VVC, AFX, and SCF exhibited the highest values, indicating superior polymerization efficiency.

## Ethics Statement

This study was approved by the Akdeniz University Faculty of Medicine Ethics Committee (Decision No. KAEK‐446, dated June 23, 2021).

## Conflicts of Interest

The authors declare no conflicts of interest.

## Data Availability

The data that support the findings of this study are available from the corresponding author upon reasonable request.
